# Mortality of Hospitalized Patients with Pleural Effusions

**DOI:** 10.4172/2161-105X.1000184

**Published:** 2014-05-01

**Authors:** Anna S Kookoolis, Jonathan T Puchalski, Terrence E Murphy, Katy LB Araujo, Margaret A Pisani

**Affiliations:** 1 Pulmonary, Critical Care Medicine, Yale University School of Medicine, USA; 2 Claude D. Pepper Older Americans Independence Center at Yale, Program on Aging, Yale University School of Medicine, USA; 3 Interventional Program (IP), Yale University School of Medicine, USA

**Keywords:** Pleural effusion, Thoracenteses, Mortality

## Abstract

**Background:**

Each year in the United States an estimated 1.5 million people develop pleural effusions and approximately 178,000 thoracenteses (12%) are performed. While it has been established that malignant effusions are associated with increased mortality, the association between mortality and all-cause pleural effusions in a medical population has not been previously evaluated. Our objective was to evaluate associations between 30-day and 12-month all-cause mortality among patients with a pleural effusion.

**Methods:**

All patients admitted to the medical service at Yale-New Haven Hospital during March 2011 were screened for pleural effusion. Pleural effusions were documented by the attending radiologist and the medical record was reviewed for admitting diagnosis, severity of illness and whether a thoracenteses was performed. The outcomes were 30-day and 12-month mortality after identification of the pleural effusion.

**Results:**

One-hundred and four patients admitted to the medical service had pleural effusions documented by the attending radiologist. At 30-days, 15% of these patients had died and by 12-months mortality had increased to 32%. Eleven (10.6%) of the 104 patients underwent a thoracenteses. Severity of illness and malignancy were associated with 30-day mortality. For 12-month mortality, associations were found with age, severity of illness, malignancy, and diagnosis of pulmonary disease. Although sample size precluded statistical significance with mortality, the hazard ratio for thoracenteses and 30-day mortality was protective, suggesting a possible short term survival benefit.

**Conclusions:**

In hospitalized medical patients with a pleural effusion, age, severity of illness and malignancy or pulmonary disease were associated with higher 12-month mortality. Thoracenteses may provide a protective effect in the first 30 days, but larger studies are needed to detect a short-term survival benefit. The presence of a pleural effusion indicates a high risk of death, with 15% of patients dying within 30 days and 32% dead within one-year of hospital admission.

## Background

The reported incidence of pleural effusions varies widely by patient population. Each year in the United States (U.S.), an estimated 1.5 million people a year develop pleural effusions. The majority of these are caused by Congestive Heart Failure (CHF), pneumonia, malignancy, and pulmonary emboli [1]. Approximately 178,000 thoracenteses are performed each year in the U.S. in an effort to assist with diagnosis or therapy [2].

The American College of Radiology recommends routine preoperative and admission chest radiographs (CXR) for older patients with stable cardiopulmonary disease who have not had a recent chest radiograph, and for persons suspected of acute cardiopulmonary disease. A CXR is useful in detecting the presence of pleural effusions. Pleural effusion volumes as low as 25 ml can be detected with erect posterior-anterior (PA) chest radiographs, whereas at least 175 ml is needed for detection with supine chest radiographs [3].

Past research has revealed an association between pleural effusions and mortality in certain patient populations. The largest prospective multicenter study evaluating the association between chest radiographs and 30-day mortality identified the presence of bilateral pleural effusions as the strongest independent predictor of mortality for patients admitted with community-acquired pneumonia (CAP). The second strongest predictor of 30-day mortality in patients with CAP was the presence of a pleural effusion classified as moderate, large, or massive [4]. Almost half of all patients with metastatic malignancies develop pleural effusions [5]. Patients with Malignant Pleural Effusions (MPE) have life expectancies ranging from 3 to 12 months, depending on the type and stage of their primary malignancy. Patients with MPE secondary to lung cancer have the shortest life expectancy, while patients with a MPE secondary to ovarian cancer have the longest life expectancy [6]. The mortality in patients with pleural effusions not associated with malignancy or acute infections is less well established.

Preliminary data from our Interventional Pulmonary (IP) program at Yale-New Haven Hospital suggests a high 30-day and one-year mortality in patients who underwent thoracenteses for pleural population of patients specifically referred for thoracenteses, or if the population with pleural effusions in general has a high mortality. In order to understand the impact of pleural effusion and thoracenteses, we performed a retrospective study to document the incidence of pleural effusions, number of thoracenteses, and the 30-day and 12-month mortality in a defined inpatient population.

## Methods

All adult patients admitted to Yale-New Haven Hospital medical floors during March 2011 who had a CXR performed within 24 hours of admission were screened for evidence of pleural effusion. Yale-New Haven Hospital is an academic teaching center with over 1000 beds. We did not include patients who were admitted to the intensive care unit, the surgical services or who were post-operative. Approval for this study was obtained by the Institutional Review Board at Yale University School of Medicine. A waiver of consent was obtained as only chart review was performed. All CXR reports were screened for the presence of a pleural effusion, as indicated by the attending radiologist. If a patient had multiple CXRs during their admission, only the first report was screened. When a pleural effusion was documented on CXR, the corresponding medical record was reviewed to obtain information about patient demographics, reason for admission, data to calculate the acute physiology and chronic health evaluation score (APACHE II), comorbid conditions, routine labs, as well as if a thoracenteses was performed during that admission. Two research assistants who underwent training and inter-rater reliability using a standard protocol for abstracting data performed chart review.

We combined reasons for hospital admission into specific groups including cardiac, pulmonary, malignancy, kidney, and other, based on the primary reason for admission as documented in the medical record. The majority of medicine patients in our hospital are referred to interventional pulmonary (IP) for thoracenteses when the procedure is deemed necessary by treating physicians. In patients who underwent thoracenteses, pleural effusions were determined to be transudative or exudative based on Light’s criteria [7]. The clinical etiologies of the pleural effusions were determined by a pulmonologist with standard criteria that has been used in previous studies [8,9]. We tracked mortality up to one year following the admission using chart review and the National Death Index. In addition, we examined the 30-day and 12-month mortality of patients in our IP database that underwent a thoracenteses for mortality comparison.

## Statistical Analysis

Descriptive statistics were calculated on patient demographics and important clinical variables and compared between persons receiving and not receiving thoracenteses. The clinical variables included reason for admission and the presence of comorbidities. Continuous variables were compared with a t-test and dichotomous variables with a chi-square statistic. A Kaplan-Meier plot of 12-month all-cause mortality was created comparing the empirical survival curves of persons with and without thoracenteses. Multivariable Cox models with fixed covariates were fit for both 30-day and 12-month all-cause mortality. In addition to thoracenteses, covariates included age, APACHE II score, gender, and indicators of three distinct reasons for admission: kidney disease, pulmonary disease, and malignancy. The associations with the reasons for admission are interpreted referent to the group comprised of patients admitted for all other conditions, including cardiac disease. All analyses were conducted using SAS V9.3 with p-values ≤ 0.05 indicating statistical significance.

## Results

**Figure 1:**
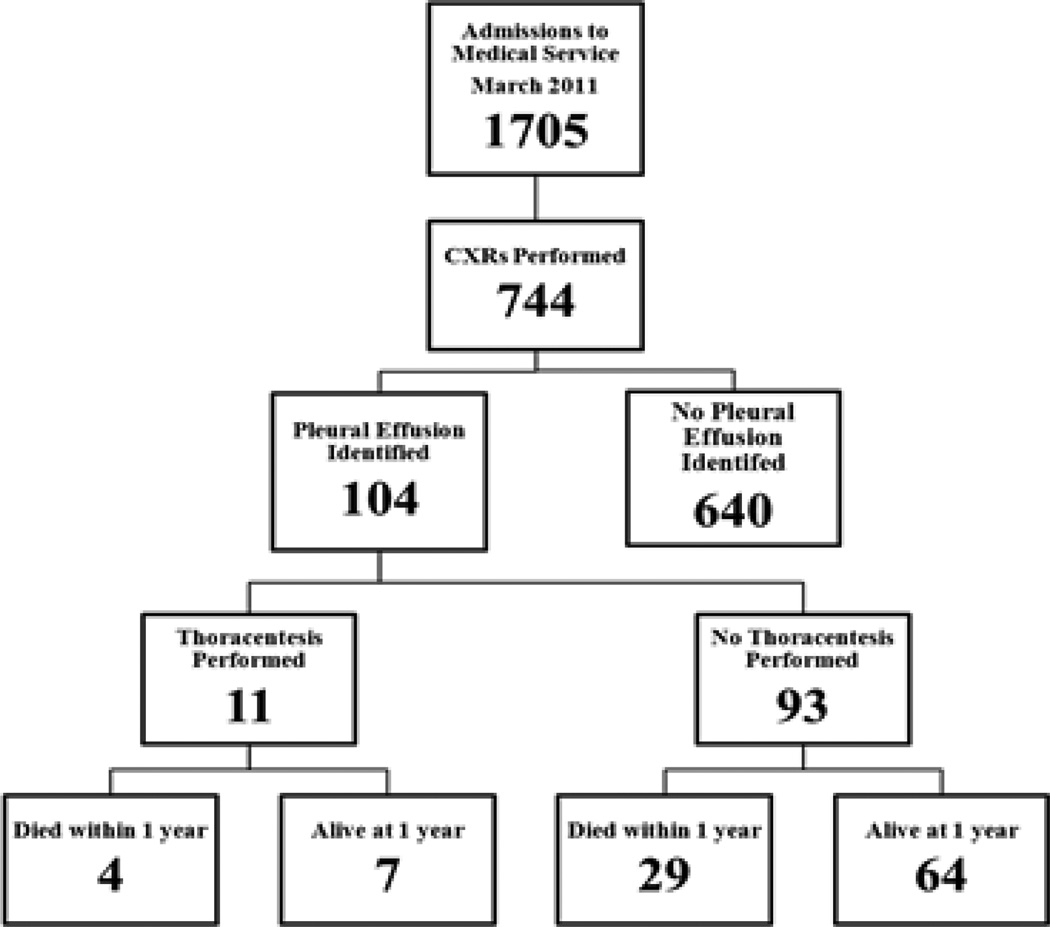
Flow diagram depicting the number of patients with pleural effusions identified on admission CXR, the number of thoracenteses performed, and the patients’ subsequent death.

Details of our study population are presented in Figure 1.

During the month of March 2011, there were 1705 patients admitted to the medical service at Yale-New Haven Hospital. Of these, 744 (43.6%), had admission CXRs performed. Pleural effusions were identified on 104 CXRs (14%). Of patients with pleural effusions, 11 underwent thoracenteses (10%). Table 1 compares demographics and medical comorbidities in patients that did and did not have thoracenteses. The mean age of the patient population was 72.7 (16.5) years. There were no significant differences in baseline demographics or reason for hospital admission between patients who underwent thoracenteses and those who did not undergo thoracenteses. Compared to other groups, patients admitted with kidney failure were less likely to have a thoracenteses performed when a pleural effusion was identified. The overall 30-day mortality in patients who had a pleural effusion identified on the admission CXR was 15%. At one year, the mortality increased to 32%.

Cardiac diseases included myocardial infarction, coronary artery disease, congestive heart failure, arrhythmia and hypertension; Pulmonary diseases included chronic obstructive lung disease, pneumonia, pulmonary embolism and obstructive sleep apnea; Malignancy included lymphoma, leukemia, lung cancer; Kidney disease included acute kidney injury; Other diseases include infection (urinary tract, line infection, peritoneal abscess), neurologic disorders such as cerebrovascular accident, intracerebral hemorrhage, hypertensive encephalopathy; gastrointestinal diseases including acute liver failure, diarrhea, dysphagia; and others such as syncope, diabetes, lower extremity thrombus, polymyositis, fever not otherwise specified, and fluid overload.

Continuous variables were tested using Student’s t-test and dichotomous variables with the chi-square statistic. There were no significant differences among the covariates between patients with and without thoracenteses.

Table 2 reports data on the etiologies of the pleural effusions in patients who had a thoracenteses performed. Nine patients underwent a unilateral thoracenteses and two patients had bilateral thoracenteses performed.

**Figure 2:**
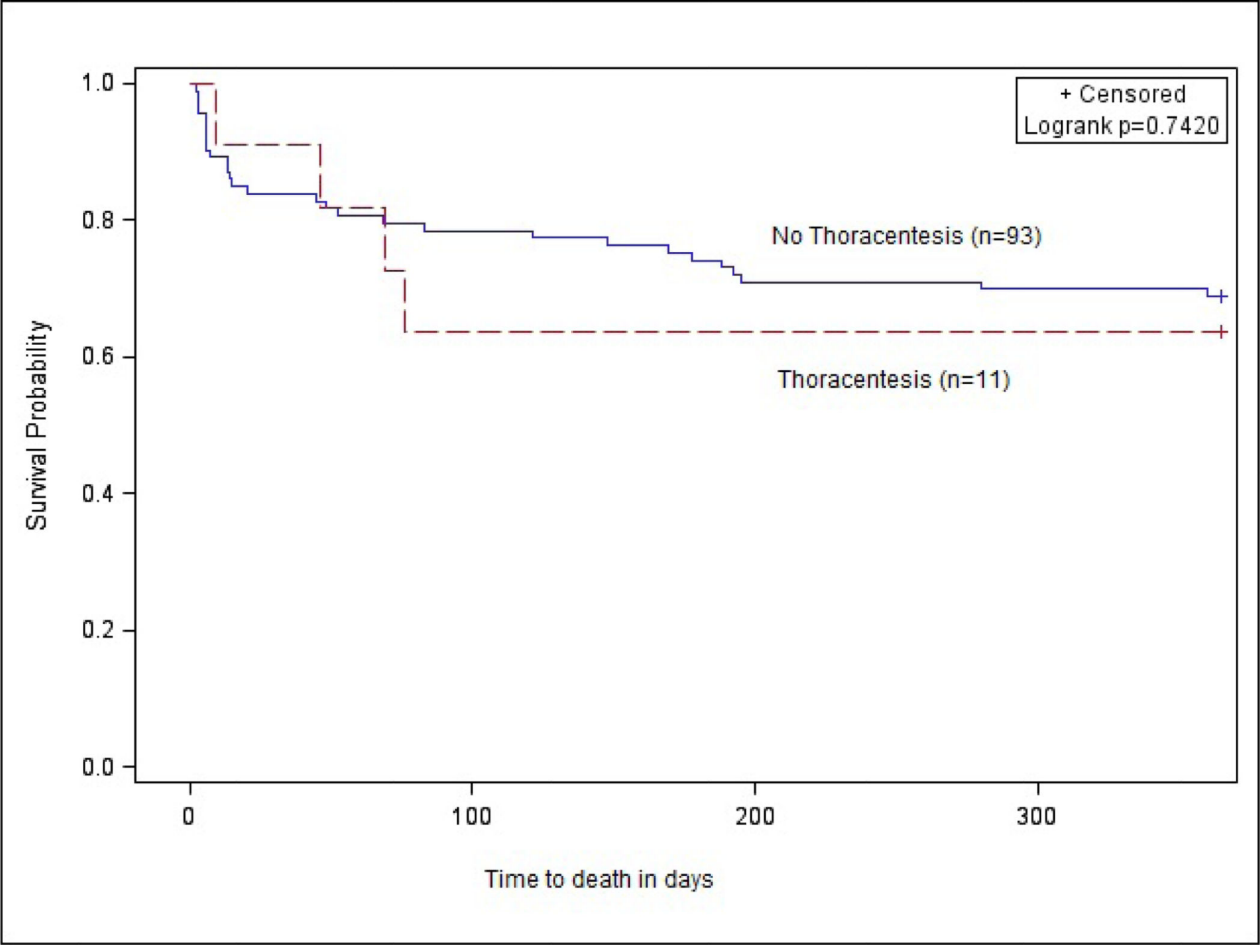
Kaplan Meier survival curve for 12-month mortality following admission with a pleural effusion (thoracenteses vs. no thoracenteses).

Figure 2 is a Kaplan-Meier plot of the 12-month survival estimates stratified by whether patients received a thoracenteses. Within the first 30 days, the survival probabilities of those who received a thoracenteses are higher. However, survival probability of those with thoracenteses drops precipitously somewhere between 50 and 100 days, remaining lower for the duration. We speculate that this reflects a short-term relief of symptoms, but a higher long term mortality reflecting the clinical fact that those receiving thoracenteses tend to be more severely ill than those not receiving it. The overall survival difference of those who did or did not receive a thoracenteses is not statistically significant over one year.

Table 3 Reports the multivariable Cox model results. The positive association between admission for cancer and 30-day mortality was statistically significant (hazard ratio [HR], 6.9; 95% CI, 1.6–29.9), as was that of APACHE II score (HR 1.1; 95% CI, 1.01–1.2). For 12- month mortality, there were significant associations with admissions for cancer (HR, 5.3; 95% CI, 1.8–15.3), pulmonary disease (HR, 2.4; 95% CI, 1.1–5.6), increasing APACHE II score (HR, 1.1; 95% CI, 1.03–1.2) and increasing age in years (HR, 1.04; 95% CI, 1.004–1.07). Although not significant for either outcome, the HR for death following thoracenteses changed from 0.51 (95% CI, 0.06 – 4.04) over 30 days to 1.34 (95% CI, 0.45 – 3.96) over 12-months. The shift from thoracenteses having a potentially protective association in the short term, to a potentially deleterious association in the longer term, is supported by the corresponding survival plots in Figure 2. A post-hoc power analysis revealed that our sample (N=104) was underpowered to detect a significant association between thoracenteses and either short term or long term mortality. We would have needed substantially higher rates of both short term and long term mortality than those observed (15% and 32%) to detect significant associations of the magnitude observed in our data.

## Discussion

We performed a retrospective chart review of all non-ICU medical admissions to Yale-New Haven Hospital during March 2011. Almost half of all patients admitted to the medical service received a CXR at the time of presentation. The prevalence of pleural effusions among patients who had a chest radiograph on admission was 14%. Not surprisingly, factors associated with increased mortality at both 30-days and 12-months were severity of illness as captured by the APACHE II score and a diagnosis of cancer. However, the overall mortality of 15% at 30 days and 32% at one year is worthy of attention.

This investigation was prompted by the mortality rate seen in patients who presented to our IP service for thoracenteses. Preliminary data from our prospective database demonstrated 35% 30-day mortality in patients with MPE, a 26% mortality in patients with multiple benign etiologies, and a 7–14% mortality in patients with CHF, liver or kidney failure. It was recognized that patients with pleural effusions presenting for thoracenteses may be different from patients who develop pleural effusions but do not undergo thoracenteses. Since our IP program only sees patients with effusions who require a thoracenteses, we had no data for comparison on patients with pleural effusions who were not referred for thoracenteses, prompting this study.

Pleural effusions are present in a large number of patients and are encountered by many medical disciplines. Whereas it is fairly well known that patients with malignant pleural effusions have a high mortality and poor prognosis, we don’t believe that physicians, in general, grasp the idea that nearly 1 in 6 patients who have a pleural effusion will die within one month of presentation, as shown by this retrospective analysis. We demonstrated that 15% of patients with a pleural effusion were dead within 30 days of admission. This increased to nearly 1 in 3 patients with a pleural effusion who were dead within 1 year of admission (31% of patients who did not undergo thoracenteses and 36% of those who did). For perspective, a recent analysis of more than 28 million hospital admissions demonstrated a 3.1% mortality for adult patients with four or more chronic medical conditions and 1.9% with 0–1 chronic medical conditions [10]. A prior study of critically ill patients demonstrated a 31% hospital mortality if patients experienced one or more organ-system failures [11].

The patients evaluated in our study had common medical problems and are likely representative of a typical inpatient population at a University hospital. Our data demonstrated that 10% of patients in the general medical inpatient population with pleural effusions underwent thoracenteses. The literature suggests that 1.5 million pleural effusions are present in the U.S. each year and that 178,000 thoracenteses are performed, indicating that 11.8% of pleural effusions are evaluated by thoracenteses. We therefore believe our population and its frequency of thoracenteses are representative of the general inpatient medical population with pleural disease.

What does this mean for our medical patients? The etiology of pleural effusions varies and the overall mortality related to these effusions likely varies by the cause of the effusion, with malignant effusions portending a poorer prognosis than benign effusions. However, the presence of a pleural effusion should call attention to the providing physician that the patient has a high 30-day and 1-year mortality. As noted above, the protective tendency with 30-day mortality exhibited by thoracenteses, and supported by the Kaplan-Meier plots, suggests that a larger scale study is needed to establish whether this protective association is statistically significant. As mentioned previously our sample size of 104 was underpowered to detect an association between thoracenteses and mortality. We note that many clinical studies are observational and underpowered, due to the well-known logistical and clinical complications of conducting research among medically ill persons [12]. The good news is that an adequately powered observational study in this population may confirm the benefit in short term mortality from thoracenteses suggested by these findings.

This study has several limitations. It is a single site study, retrospectively performed over a one month period. There may be seasonal variations in pleural fluid development that may relate to the underlying etiology of the effusion. We may have underestimated the numbers of effusions if a patient never received a chest radiograph that documented pleural effusion. An additional limitation is that the specific cause of death for these patients was not identified. For instance, a patient with an effusion due to pneumonia may have actually died from myocardial infarction or a trauma in the subsequent year. Rather, our results suggest that persons with pleural effusions constitute a patient population that appears to be at considerable risk for death.

Strengths of this study include the general applicability to a general inpatient medical population. We performed a systematic chart review and analyze a current gap in the current research that has focused previously on critically ill patients or those with malignancy and infection. We reviewed a large number of admissions and CXRs performed. We propose that the presence of a pleural effusion identified may serve as a “red flag” for physicians caring for these patients, and that its presence may signify the need for a stronger alert signal in the medical records, just as lab abnormalities are highlighted when critical results are present. The major novelty of this study is its provision of evidence-based information confirming that on average, the mere presence of a pleural effusion indicates considerable risk of both short and long term mortality.

## Conclusion

The majority of patients admitted to general medical services have conditions that may be associated with pleural effusions. Many patients with pleural effusions die within 30-days of admission to the hospital, and nearly 1/3 are dead within one year. A higher level of aggressive medical therapy may be warranted for those patients who present with pleural effusions in order to decrease their potential risk of death. Pleural effusions may serve as a marker of mortality in this patient population.

## Figures and Tables

**Table 1:** Participant Characteristics by Thoracenteses (N=104).

	No Thoracenteses n=93	Thoracenteses n=11
Participant Characteristics		
Age in years, mean (SD)	73.2 (16.6)	68.4 (15.9)
Female gender, n (%)	51 (55)	7 (64)
Nonwhite race, n (%)	20 (22)	2 (18)
BMI>30, n (%)	21 (24)	2 (20)
APACHE II, mean (SD)	11.1 (5.5)	10.9 (3.7)
Admission Reason, n (%)		
Cardiac disease	32 (34)	3 (27)
Pulmonary disease	24 (26)	5 (45)
Malignancy	8 (9)	2 (18)
Kidney disease	7 (8)	0 (0)
Other	14 (23)	1 (9)
Medical Comorbidities, n (%)		
Cardiac disease	75 (81)	9 (82)
Pulmonary disease	33 (35)	2 (18)
Malignancy	32 (34)	4 (36)
Kidney disease	28 (30)	1 (9)
30 Day Mortality, n (%)	15 (16)	1 (9)
12 Month Mortality, n (%)	29 (31)	4 (36)

APACHE II-Acute Physiology and Chronic Health Evaluation

**Table 2:** Characteristics of Pleural Fluid in Patients Undergoing Thoracenteses (N=11).

Characteristics of Pleural Fluid in Patients Undergoing Thoracenteses (N=11)	n (%)
Unilateral	9 (82)
Bilateral	2 (18)
Etiologies of pleural effusion	
Malignancy	2 (18)
Congestive heart failure (CHF)	1 (9)
Malnutrition	1 (9)
Hepatic hydrothorax	1 (9)
Multiple etiologies*	4 (37)
Pericarditis	1 (9)
Unknown	1 (9)

* Multiple etiologies included a combination of CHF/Kidney failure, CHF/Kidney failure/Pneumonia, CHF/Rib Fracture, Liver failure/Pneumonia

**Table 3:** Multivariable Associations with Mortality after Diagnosis of Pleural Effusion

30-day Mortality (N= 104 with 16 deaths)		
Variable	HR (CI)*	P Value
APACHE II	1.10 (1.01–1.22)	0.04**
Age, years	1.01 (0.97–1.05)	0.72
Male	0.60 (0.18–2.00)	0.21
Thoracenteses performed	0.51 (0.06–4.04)	0.52
Admission Reason:		
Pulmonary disease	2.55 (0.65–10.05)	0.18
Malignancy	6.94 (1.61–29.86)	0.01**
Kidney disease	1.41 (0.15–12.89)	0.76
12 month Mortality (N = 104 with 33 deaths)		
APACHE II	1.11 (1.03–1.19)	0.01**
Age, years	1.04 (1.01–1.07)	0.03**
Male	0.79 (0.38–1.66)	0.54
Thoracenteses performed	1.34 (0.45–3.96)	0.60
Admission Reason:		
Pulmonary disease	2.45 (1.06–5.64)	0.04**
Malignancy	5.27 (1.82–15.31)	0.01**
Kidney disease	0.89 (0.20–4.06)	0.88

HR-hazard ratio; CI- 95% confidence interval; APACHE-Acute Physiology and Chronic Health Evaluation;

* HR and CI calculated from Cox Proportional Hazards Model

** Statistical significance defined as a p-value < 0.05.
